# Understanding the inhibitory mechanism of tea polyphenols against tyrosinase using fluorescence spectroscopy, cyclic voltammetry, oximetry, and molecular simulations†

**DOI:** 10.1039/c7ra12749a

**Published:** 2018-02-22

**Authors:** Haifeng Tang, Fengchao Cui, Haijuan Li, Qingrong Huang, Yunqi Li

**Affiliations:** Key Laboratory of Synthetic Rubber, Changchun Institute of Applied Chemistry (CIAC), Chinese Academy of Sciences Changchun Jilin 130022 P. R. China fccui@ciac.ac.cn yunqi@ciac.ac.cn; State Key Laboratory of Electroanalytical Chemistry, Changchun Institute of Applied Chemistry (CIAC), Chinese Academy of Sciences Changchun Jilin 130022 P. R. China; School of Life Science, Jilin University Changchun Jilin 130012 P. R. China; Department of Food Science, Rutgers University 65 Dudley Road New Brunswick NJ 08901 USA; University of Chinese Academy of Sciences Beijing 100049 P. R. China

## Abstract

Inhibiting the activity of tyrosinase is a very effective and safe way to prevent enzymatic browning in food and to resist pests in agriculture. Tea polyphenols (TPs), regarded as safe and non-toxic food additives, have been reported due to their potential inhibitory capability against tyrosinase, but their ambiguous inhibitory mechanisms have severely limited their application. In the present work, fluorescence spectroscopy, cyclic voltammetry (CV), oximetry and molecular simulation approaches were employed to shed light on the underlying inhibitory mechanisms of TPs with different structures including (+)-catechin, (−)-epicatechin gallate (ECG) and (−)-epigallocatechin gallate (EGCG) against tyrosinase. Fluorescence spectra show that the three TPs are capable of binding tyrosinase with a molar proportion of 1 : 1. The analysis of CV curves and oxygen utilization suggests that these three TPs can be oxidized by tyrosinase, revealing that these three TPs are suicide inhibitors of tyrosinase. Furthermore, ECG and catechin make tyrosinase irreversibly inactivated due to their catechol group (ring B) being catalyzed by tyrosinase through a cresolase-like pathway, while EGCG inhibits the activity of tyrosinase by competing with or delaying the oxidation of substrate. Molecular simulations further confirm that ring B of ECG and catechin makes a significant contribution to tyrosinase inhibitory activities, and has a direct interaction with the coupled binuclear copper ions in the optimal orientation required by the cresolase-like pathway.

## Abstract 2

Revealing the inactivation mechanism of TPs on inhibiting the activity of tyrosinase.

## Graphical abstract

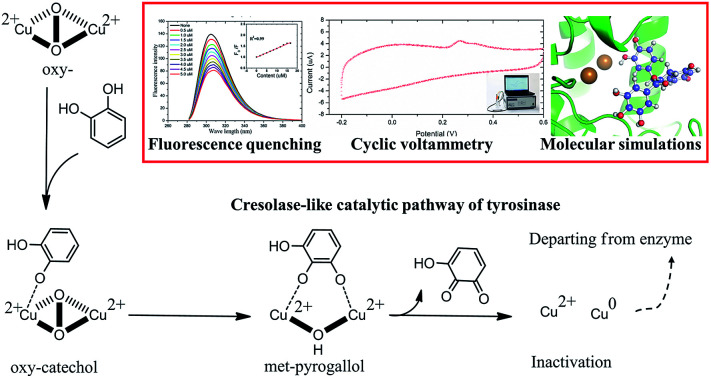

## 1. Introduction

Tyrosinase (EC 1.14.18.1) containing a catalytic center with bi-copper ions coordinated by six histidine residues, can catalyze the oxidation of both monophenol (cresolase or monophenolase activity) and diphenol (catecholase or diphenolase activity) to the corresponding *ortho*-quinones, followed by self-polymerizing to dark melanin or another pigment.^1–4^ In these oxidation reactions, the redox forms of the catalytic center include Cu_2_–O_2_ with both monophenolase and diphenolase activities, Cu_2_–O with diphenolase activity and Cu_2_ without any activity, according to the amount of coordinated oxygen.^1^ In the food industry, tyrosinase can expedite the enzymatic browning of post-harvest produce by catalyzing the oxidation of polyphenol compounds,^4^ leading to the loss of nutritional quality and the shortening of storage life.^5^ Moreover, the higher tyrosinase activity would adversely affect the pest control in agriculture due to promoting the physiological processes of larval maturity.^6,7^ Inhibiting tyrosinase bioactivity is therefore favorable to the preservation of agricultural product and pest prevention. Although a plenty of natural tyrosinase inhibitors have been discovered, the understanding of the inhibition mechanism of tyrosinase inhibitors is still deficient, which have severely limited their application in food industry and agriculture and the development of new inhibitors.^8–11^

Flavonoids, widely used as dietary supplements to functional foods,^12^ are the largest groups in natural tyrosinase inhibitors which have been discovered,^13–17^ up to now. Flavonoids were usually subdivided into six major groups including flavanols, flavones, flavonols, flavanones, isoflavones and anthocyanidins.^18^ The position and number of hydroxyl group on functional moiety of flavonoids have a significant effect on their inhibitory activity and associated mechanism. Some flavonols with a 3-hydroxyl-4-keto moiety, such as kaempferol, quercetin and morin, exhibit the inhibitory activity toward tyrosinase by chelating with the copper ions and occupying the catalytic center,^15,19^ but most of flavonols have weaker inhibitory activity than kojic acid.^20^ Two isoflavone metabolites (8-hydroxydaidzein and 8-hydroxygenistein), deemed as the suicide substrate of tyrosinase, can make tyrosinase irreversibly inactivated by substrate-like interaction.^14^ Tea polyphenols (TPs), such as (+)-catechin (catechin), (−)-epicatechin gallate (ECG) and (−)-epigallocatechin-3-*O*-gallate (EGCG), which belong to flavanols, frequently present in human diet due to their strong antioxidant activity. The report has shown that they can competitively inhibit the monophenolase activity of tyrosinase *in vitro*.^21^ Particularly, EGCG can penetrate the cell membrane and suppress the activity of tyrosinase in B16 murine melanoma cell.^22^ Although the inhibitory activity of these TPs against tyrosinase has been reported, their inhibitory mechanisms are still elusive.

**Fig. 1:**
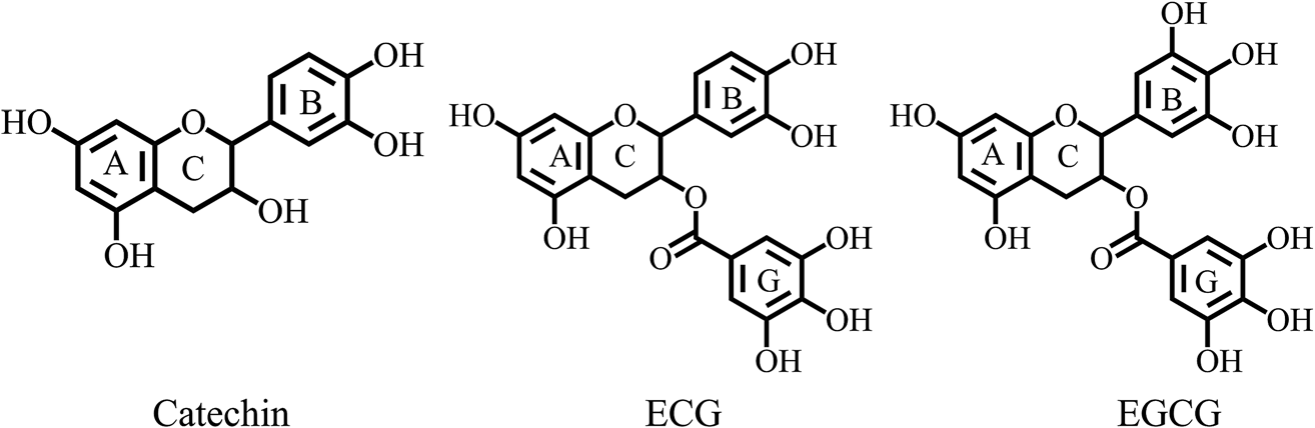
Molecular structures of TPs.

In this work, we managed to reveal the inhibitory mechanisms of TPs (including catechin, ECG and EGCG as depicting in Fig. 1) using fluorescence spectroscopy, cyclic voltammetry (CV) and oximetry together with molecular simulations. Fluorescence spectra determined the nature of interactions between TPs and tyrosinase. CV identified the inhibitory mechanism of TPs with different functional moieties. Oxidation kinetics of tyrosinase discovered the inactivation of TPs against tyrosinase by Quintox mechanism. Molecular simulations provided a deep understanding of their inhibitory mechanism against tyrosinase at the molecular level.

## 2. Materials and methods

### 2.1 Chemical reagents and apparatus

Tyrosinase (TYR, EC1.14.18.1) from mushroom, l-3,4-dihydroxyphenylalanine (l-DOPA), kojic acid (KA), catechin, ECG, and EGCG were purchased from Sigma-Aldrich. Nitric acid and dimethyl formamide (DMF) were purchased from Beijing Chemical Works. Multiwalled carbon nanotubes (MWCNTs) were purchased from the Nanotech Port Co. Ltd (Shenzhen, China). Before use, the MWCNTs were acidified by nitric acid.^23^

Spectrophotometer reader (BioTek Instruments Inc., USA) and High Performance Liquid Chromatograph (HPLC: Shimadzu Instruments Inc., Japan) were used to measure the inhibitory activity of the tyrosinase inhibitors. CV experiments were performed in a standard three-electrode electrochemical cell with a CHI 660E electrochemical analyzer (CH Instruments, Chenhua Co., Shanghai, China). Oxygen consumptions of tyrosinase interacting with TPs were monitored with YSI 5000 dissolved oxygen meter (YSI Inc., American).

### 2.2 Inhibitory activity *in vitro*

The inhibitory activity of the TPs, *i.e.*, catechin, ECG, and EGCG, and a positive control, *i.e.*, KA, against diphenolase activity of tyrosinase was measured with l-DOPA as a substrate using the reported spectroscopy method.^13,24^ Assays were conducted in a 96-well micro-plate and a spectrophotometer reader was used to determine the absorbance.^25^ Briefly, 6 μL of 1000 unit per mL tyrosinase solution was combined with potassium phosphate buffer (pH 6.5) and 1 mM KA or TPs (both dissolved in potassium phosphate buffer, pH 6.5). After pre-incubating at 25 °C for 5 min, 150 μL of 1 mM l-DOPA aqueous solution was added, followed by incubation at 25 °C for 10 min, again, before the concentration determination of DOPA quinone at 475 nm absorbance. The total volume of each reaction system was 300 μL. As an exception, the tyrosinase inhibitory activity of catechin was measured with HPLC rather than spectroscopy, as it possessed an overlap adsorption band with DOPA quinone which will interfere the analysis of inhibitory activity.^26^ The inhibitory activity was expressed by IC_50_ value, which is equal to the concentration of inhibitor at 50% inhibitory rate according to the following equation:^25^1
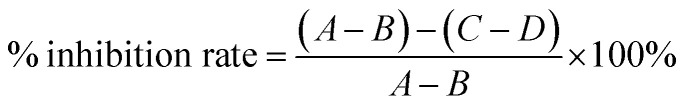
where, *A* is the optical density (OD_475_) of potassium phosphate buffer; *B*, *C* and *D* are the OD_475_ of potassium phosphate buffer with tyrosinase, with both TPs and tyrosinase, and with TPs, respectively.

### 2.3 Fluorescence spectroscopy

The fluorescence spectrum of the TP-TYR complex was measured according to our previous work.^27^ Briefly, 10 mM TP was titrated into 3 mL potassium phosphate buffer (pH 6.5) containing 10 units tyrosinase under continuous stirring and nitrogen exists. After 5 minutes, tyrosinase was excited at 274 nm and the emission spectrum over the range of 280 nm to 400 nm was detected through a 3 nm slit. The emission spectrum of the tyrosinase solution was also directly measured as a background.

### 2.4 Cyclic voltammetry

The acidified MWCNT was suspended in DMF at a concentration of 200 mg mL^−1^, and then 5 μL of this dispersion was dropped onto the polished surface of the GC electrode and allowed to dry naturally for 12 h. The dried electrode was submerged in 3 mg mL^−1^ tyrosinase dissolved in 10 mM potassium phosphate buffer (pH 4.5) for 24 h.

CVs were performed in an electrolyte of 10 mM TP and 10 mM potassium phosphate buffer (pH 6.5) in the range from −0.2 V to 0.4 V at a scan rate of 0.05 V s^−1^.

### 2.5 Oximetry

The oxygen consumption in the process of tyrosinase interacting with TPs was monitored with the reported method.^28^ Briefly, 0.4 mL of 8.2 mM TP was added to 19.2 mL phosphate buffer (pH 6.5). After equilibrating at 30 °C for 5 min, 0.4 mL of 500 unit per ml tyrosinase was injected to TP solution under continuous stirring. The decrease of oxygen was monitored with the dissolved oxygen meter. In order to evaluate the remaining activity of tyrosinase, l-DOPA was added to the above mixture of tyrosinase and TP after 5 minutes if no oxygen decreasing was observed, followed by monitoring the secondary oxygen utilization in the oxidation reaction of l-DOPA catalyzed by the remaining tyrosinase. The concentration of l-DOPA was 194 μM.

### 2.6 Molecular simulations

The TP structures were generated using Molinspiration Galaxy 3D Structure Generator v. 2013.02 beta (https://www.molinspiration.com) followed by optimization using B3LYP^29–31^ with a 6-31G (d, p) basis set implanted in Gaussian 09. The crystal structure of tyrosinase with tropolone (PDB ID: 2Y9X)^32^ was taken from the Protein Data Bank (PDB).^33^ Ligands and crystal water molecules were removed and missing hydrogen atoms were added using Schrödinger software.^34^ The protonation and disassociation of chargeable residues (His, Asp, Glu, Arg, and Lys) and N- and C-terminus residues were set at pH 7.0.^35,36^ Partial charges in the six histidine residues (60, 84, 93, 258, 262, and 295) and two copper ions in the catalytic center were set according to the report of Choi *et al.*^37^ All residues of the protein were parameterized with the AMBER-03 force field.^38^ The cluster of bi-copper ions coordinated by six histidine residues were firstly optimized using B3LYP with a 6-31G (d, p) basis set, and then the parameters of bonds, angles and dihedral formed between the histidine residues and copper ions were evaluated using the Visual Force Field Derivation Toolkit (VFDT).^39^ A semi-flexible docking was performed by AutoDock Vina^40^ to identify binding poses of TPs with tyrosinase, where tyrosinase was treated as a rigid body and all rotatable bonds in the ligand were sampled. Optimal binding sites were searched in a grid box of 30 × 30 × 30 Å^3^ centered on the bi-copper center of tyrosinase.

The optimal binding modes were further refined with fully flexible atomic molecular dynamics (MD) simulation using NAMD (v. 2.9).^41^ The binding free energy of all TPs with tyrosinase was evaluated using the Molecular Mechanics/Poisson–Boltzmann Surface Area (MM/PBSA) method^42^ and was further decomposed into the van der Waals (Δ*E*_vdW_), electrostatic (Δ*E*_ele_), nonpolar solvation (Δ*G*_nonpolar_) and polar solvation (Δ*G*_polar_). The conformational entropy (−*T*Δ*S*) was calculated by a normal-mode analysis.^43^ More detailed simulation information can be found in our previous papers.^44,45^

## 3. Results and discussion

### 3.1 Tyrosinase inhibitory activities of TPs

The IC_50_ value of the TPs and KA (acted as positive control) was measured with l-DOPA as a substrate and listed in Table 1 and the profile was depicted by ESI Fig. 1.† These natural compounds can inhibit diphenolase activity of tyrosinase with dose-dependent manner and their inhibitory activities against tyrosinase ranked by IC_50_ values were: ECG > KA > catechin > EGCG. The structural difference between ECG and EGCG is less of hydroxyl group on ring B of ECG, the tyrosinase inhibitory activity of ECG was 6-fold stronger than that of EGCG. This suggested that the catechol group (with two hydroxyl group) on the TPs was crucial in enhancing its tyrosinase inhibitory activity. We speculated that the catechol group (ring B) should have a direct interaction with a coupled binuclear copper site in the catalytic center. Moreover, we found that the pyrogallol group (ring G) would be favorable to the tyrosinase inhibitory activity of TPs by comparing ECG and catechin. The important role of the pyrogallol group in tyrosinase inhibitory activity of compounds has also been reported by Kim and Uyama.^18^

**Table 1:** The measured IC_50_ value and Stern–Volmer quenching constant (*K*_SV_), bimolecular quenching constant (*κ*_q_), binding constant (*K*_A_) and binding number (*n*) obtained from fluorescence spectra

Inhibitors	IC_50_ (μM)	*K*_SV_ (×10^4^ M^−1^)	*κ*_q_ (×10^12^ M^−1^ s^−1^)	*K*_A_ (×10^5^)	*n*
Catechin	57.12	0.48	0.48	0.04	0.99
ECG	22.63	4.10	4.10	0.53	1.03
EGCG	142.40	3.13	3.13	3.07	1.23
KA	35.70	NA	NA	NA	NA

### 3.2 Fluorescence quenching

**Fig. 2:**
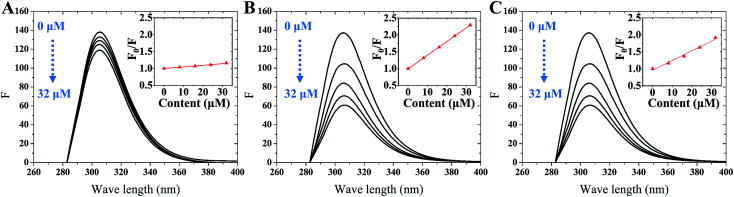
Emission spectra of tyrosinase excited at 274 nm wavelength in the presence of different concentrations of catechin (A), ECG (B) and EGCG (C). Inset: Stern–Volmer plots describe the tyrosinase quenching caused by binding with TPs.

Fluorescence spectroscopy was used to further detect the interaction between tyrosinase and TPs by monitoring the quenching of the intrinsic residues in tyrosinase. The fluorescence emission spectra of tyrosinase excited at 274 nm wavelength were collected with different concentration gradients of TPs and depicted in Fig. 2. It can be observed that the fluorescence intensity of tyrosinase decreased gradually with the increase of the concentration of TPs, indicating that TPs could interact with tyrosinase and quench the fluorescence of residues. Fig. 2 shows a strong 305 nm fluorescence emission peak of tyrosinase in each TP aqueous solution, and there is no significant shift in the fluorescence emission peak of tyrosinase with either the increase of TPs concentration or the addition of other TP molecules.

Fluorescence quenching mechanism is usually divided into static or dynamic manner.^46^ To clarify the quenching mechanism of the interaction between tyrosinase and TPs, we analyzed the fluorescence quenching data using the Stern–Volmer equation.^47^2
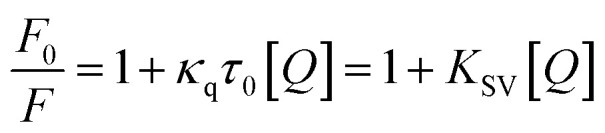
where *F*_0_ and *F* are the relative fluorescence intensities before and after the addition of inhibitor, respectively; *κ*_q_ is the bimolecular quenching rate constant; *τ*_0_ is the fluorescence lifetime at 10^−8^ s for most biological macromolecules,^48^ [*Q*] is the concentration of inhibitor and *K*_SV_ is the Stern–Volmer quenching constant. The Stern–Volmer quenching constant (*K*_SV_) determined by linear regression of a curve of *F*_0_/*F versus* [*Q*] was plotted in Fig. 2 (inset). As listed in Table 1, the *K*_SV_ value of ECG with less one hydroxyl group on ring B is much higher than that of EGCG. Good linear relationships in three Stern–Volmer plots of TPs are generally the indication of a single type of quenching manner. The calculated *κ*_q_ values by *K*_SV_/*τ*_0_ are 0.48 × 10^12^, 4.10 × 10^12^ and 3.13 × 10^12^ M^−1^ for catechin, ECG and EGCG, respectively. The *κ*_q_ values of tyrosinase quenching procedure caused by three TPs were higher than the limiting diffusion collision quenching constant of the biomolecules (2.0 × 10^10^ M^−1^ s^−1^) by two order of magnitude, suggesting that the quenching process of tyrosinase did not follow dynamic manner,^49^ but was a static one.^48,50^ For static quenching mechanism, the binding site number (*n*) and association constant (*K*_A_) can be obtained by the following equation:^49^3
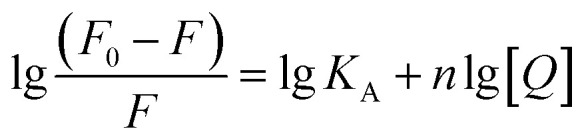


The binding sites number of each TP with tyrosinase approximately equals to 1 (ESI Fig. 2†), which indicates that one tyrosinase could only be bound by one TP molecule. However, the calculated association constants (*K*_A_) are in the order of EGCG (3.07 × 10^5^) > ECG (0.53 × 10^5^) > catechin (0.04 × 10^5^), being significant inverse relation with their inhibitory activities. This may suggest that the inhibitory mechanism of TPs on tyrosinase is not apparently competitive with its substrate binding into the catalytic site of tyrosinase.

### 3.3 Inhibitory mechanism of TPs

**Fig. 3:**
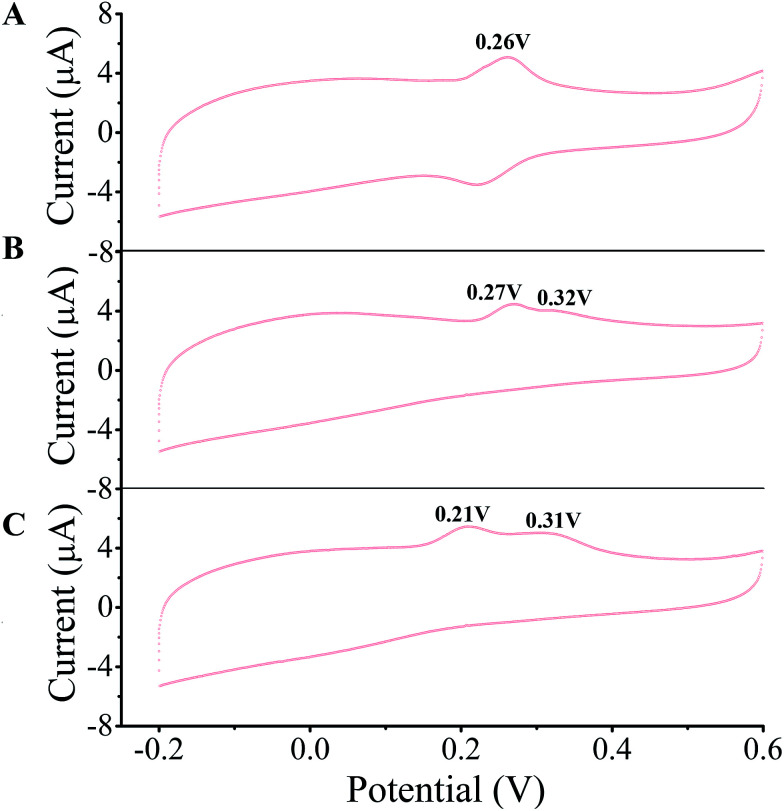
Cyclic voltammetry (CV) curves of 20 μM catechin (A), ECG (B) and EGCG (C) measured on glassy carbon electrode modified by MWCNT and tyrosinase at 50 mV s^−1^ in pH 6.5 phosphate buffer. The oxidation peaks are marked.

A few of investigations have reported^51–55^ that flavonoids inhibited tyrosinase activity either by chelating with the coupled bi-copper ions or by acting as substrate of tyrosinase. To deeply understand tyrosinase inhibitory mechanism of TPs, belonging to flavonoids, we traced the fluctuation of oxidized current accompanied by the addition of TPs into tyrosinase solution using the cyclic voltammetry (CV) approach, which has been extensively applied to determine the antioxidant potential of flavonoids.^56–59^Fig. 3 depicts the CV curves of catechin, ECG and EGCG. The presence of significant current peaks in the CV curves indicates that three TPs should be oxidized by tyrosinase as its substrate. The catechin has always been used as a substrate of tyrosinase.^51,60–62^ Thus, we speculated that three TPs could interact with tyrosinase with substrate-like manner and inhibit tyrosinase activity by reducing tyrosinase. As shown in Fig. 3, two peaks were detected for ECG and EGCG, while for catechin only one peak was found. The peak at 0.26 V in the CV curve of catechin should arise from the oxidization of the catechol group (ring B with *ortho*-diphenols) catalyzed by tyrosinase, because *meta*-diphenols, *i.e.* ring A, have been found to have higher oxidation potentials than *ortho*-diphenols.^56^ Kilmartin *et al.*^57^ reported that the oxidization peak of *meta*-diphenols on ring A of flavonoids (such as catechin) was higher than 0.7 V. By comparing the CV curves of ECG with the one of catechin, it can be concluded that the first peak of ECG (0.27 V) corresponds to the oxidation of the catechol group (ring B), contained by both catechin and ECG. The difference between the CV curves of ECG and EGCG can identify that the second peak originates from the oxidation of the pyrogallol group (ring G) of both ECG and EGCG, while the first peak of EGCG (0.21 V) is caused by the oxidation of its pyrogallol group (ring B with three hydroxyl groups). The lower oxidization peak potential of the pyrogallol group compared with the catechol group has also been found by Furuno.^63^ It should be noted that the first peak of ring B of EGCG has a much lower oxidation potential than that of ECG, indicating it should possess much stronger reductive activity. Thus, the order of ability in reducing tyrosinase should be EGCG > catechin ≈ ECG. However, this order is inversely correlated with their order of tyrosinase inhibitory activity. One possible explanation is that the oxidation of catechin, ECG and EGCG catalyzed by tyrosinase should undergo different reaction pathways, leading to the different inhibitory mechanisms.

**Fig. 4:**
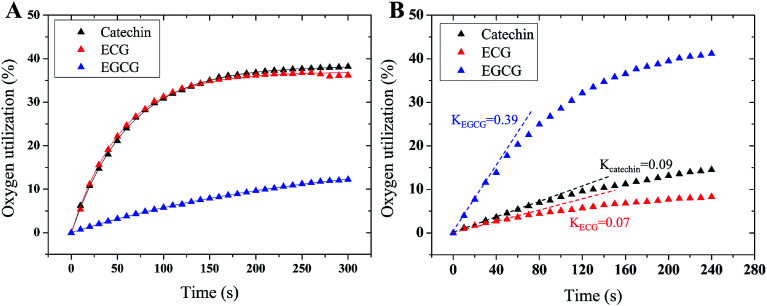
The time-dependent curves of oxygen utilization in the oxidation of (A) TPs catalyzed by tyrosinase and (B) l-DOPA catalyzed by the remaining tyrosinase. The initial reaction velocities of tyrosinase in oxidizing l-DOPA are illustrated by the slope of oxygen utilization curves at the starting reaction time.

**Fig. 5:**
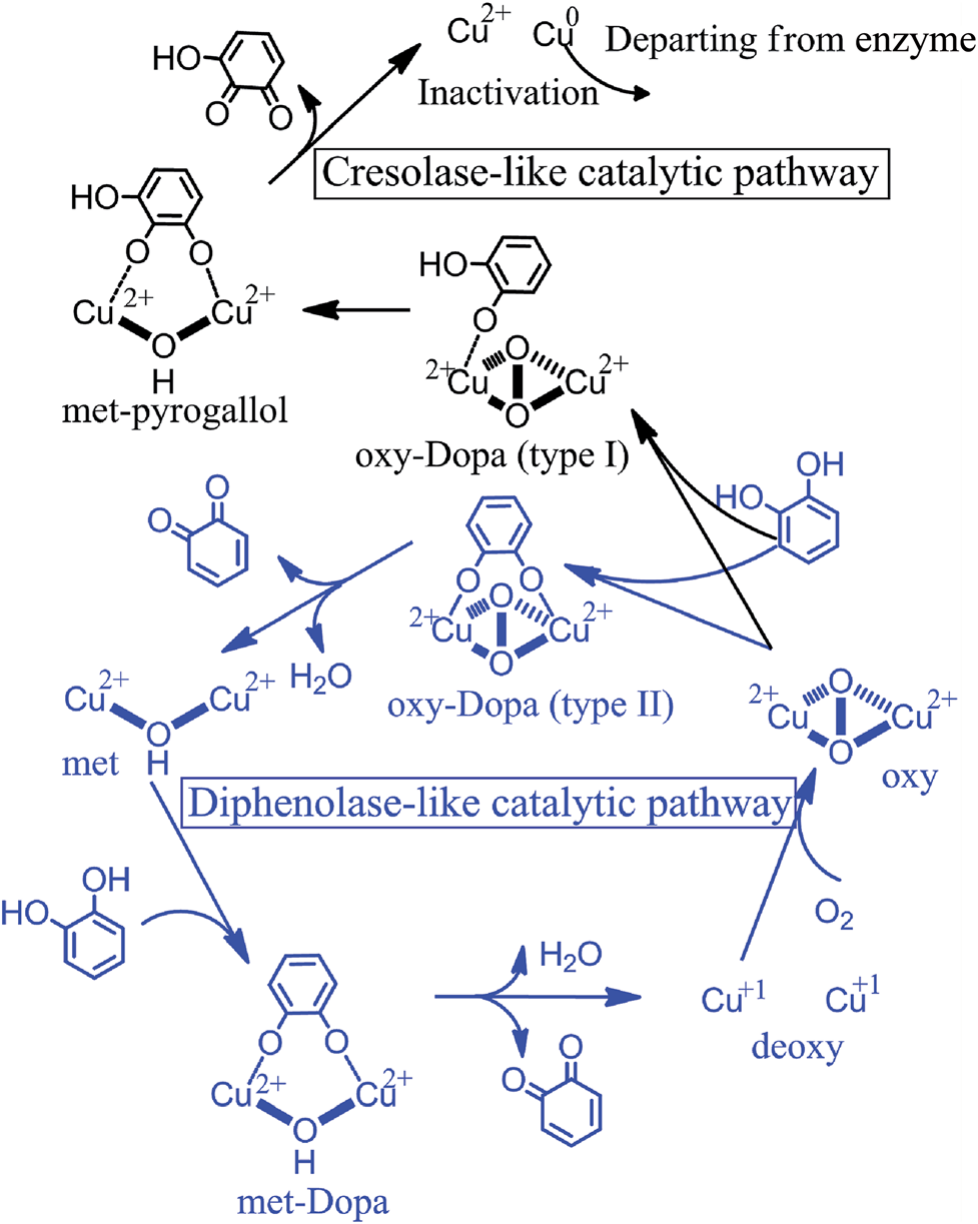
Cresolase-like and diphenolase-like catalytic pathways of tyrosinase, which catalyze the monophenols (black) and diphenols (blue), respectively. Both catalytic mechanisms were reported by Solomon *et al.* and Land *et al.* and re-depicted here.

To further reveal tyrosinase inhibitory mechanisms of TPs, we monitored oxygen utilization against time starting from the injecting of TPs into tyrosinase solution using oximetry. The corresponding time-dependent curves of oxygen utilization are depicted in Fig. 4A. In the initial stage, the increase of oxygen utilization for TPs further corroborates that TPs could be oxidized by tyrosinase like-as substrate manner. The time-dependent curves of oxygen utilization can be better fitted with the following equation:^28^4
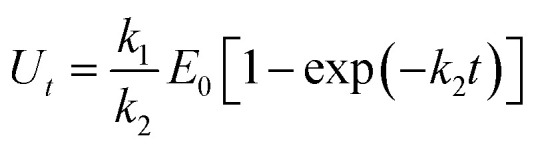
where, *U*_*t*_ is the oxygen consumption at time *t*; *E*_0_ is the initial amount of tyrosinase; *k*_1_ and *k*_2_ are the oxidation rate and the inactivation rate of tyrosinase, respectively. As listed in Table 2, ECG and catechin can inactivate tyrosinase with inactivation rate (*k*_2_) of 0.018 and 0.016, respectively, while the inactivation rate of EGCG is ∼4 times lower than catechin. The order of tyrosinase inactivation rate by TPs is in according with their inhibitory activity, indicating that whether inactivating tyrosinase more or not is the vital factor for the inhibitory activity of TPs. Furthermore, the catecholase activities of residual tyrosinase were evaluated by the oxygen utilization of l-DOPA. Fig. 4B displays the curves of oxygen utilization against time in the oxidation reaction of l-DOPA catalyzed by the residual tyrosinase in each TP solution. The slope (*K*) of oxygen consumption curve is positive proportional to the amount of residual tyrosinase.^64^ Obviously, the remaining quantity of tyrosinase for EGCG is largest, while the residual amount is least for ECG. This indicates that the catechol group (ring B with *ortho*-dihydroxy groups) on ECG or catechin plays crucial roles in inhibiting tyrosinase activity. Therefore, we proposed that the catechol group (ring B) of ECG and catechin resulted in the inactivation of tyrosinase by reducing tyrosinase with Quintox mechanism through cresolase-like catalytic pathway, as reported by Land *et al.*^28^ In this inactivation pathway, the catechol is converted to an intermediate by deprotonation and reductive elimination along with the leaving of forming Cu(0) from catalytic center, leading to the irreversible inactivation of tyrosinase.^28^ However, the oxidation of ring B on EGCG or ring G on both ECG and EGCG (the pyrogallol group) catalyzed by tyrosinase should be competitive with the natural substrate (such as l-DOPA) through the diphenolase-like catalytic pathway (as shown in Fig. 5), because the three adjacent hydroxyl groups on the pyrogallol group having larger steric hindrance and worse orientation were unfavorable to the insert of additional oxygen on the aromatic ring.^28^ Tyrosinase at last can be reactivated after catalyzing the oxidation of two molecules of EGCG, accounting for the lower inhibitory activity of EGCG than ECG and catechin. The partition ratio (*r*), which is the molar proportion of inhibitors and inactive enzyme, is usually used to describe the inactivation capability of substrate with inactivating ability.^14,65^ As shown in ESI Fig. 3† (the intercept on abscissa), inactivating one molecule of tyrosinase requires 14.7 molecules of ECG or 38.7 molecules of catechin, being better consistent with their inactivation rate. The lower inactivation efficiency against tyrosinase explained that catechin has weaker inhibitory activity than ECG. Overall, three TPs inhibit the activity of tyrosinase with different inhibitory mechanisms: the catechin inactivates tyrosinase irreversibly with lower efficiency; EGCG inhibits tyrosinase activity by competing with or delaying the oxidation of substrate; ECG possesses both two inhibitory mechanisms and higher inactivation efficiency against tyrosinase.

**Table 2:** The oxidation rate (*k*_1_), inactivation rate (*k*_2_) of TPs against tyrosinase and the slope (*K*) of the residual tyrosinase oxidizing l-DOPA substrate

Inhibitors	*k*_1_	*k*_2_	*K*
Catechin	0.063	0.016	0.009
ECG	0.068	0.018	0.007
EGCG	0.007	0.004	0.390

### 3.4 Binding behaviors of TPs with tyrosinase

**Fig. 6:**
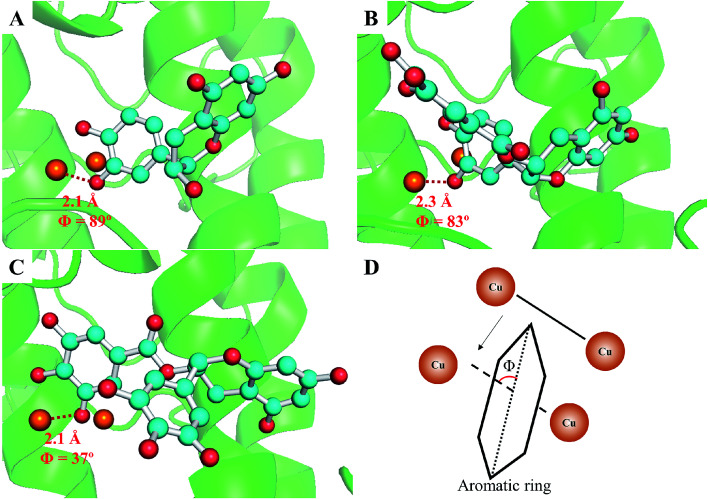
The optimal binding structures of catechin (A), ECG (B) and EGCG (C) with tyrosinase clustered out from MD simulation trajectories. The dicopper ions in the catalytic center are represented as orange spheres, tyrosinase and the skeleton of TPs are shown using cartoon and ball-and-stick model, respectively. The included angles between the ring B of TPs and the connected line of two copper ions (illustrated in D) and the distances between the oxygen atoms of ring B and copper ions were labeled.

To further confirm the different inhibitory mechanisms of TPs at the molecular level, we investigated the binding behaviors of TPs with tyrosinase using molecular docking, molecular dynamics simulations and MM/PBSA calculations. The binding mode of TPs was determined by molecular docking. As depicted in ESI Fig. 4,† all functional groups (ring A, B and G) of three TPs can approach to the catalytic center of tyrosinase containing bi-copper ions. Based on the optimal binding conformation for each functional group, we performed 30 ns MD simulations to refine the structure of TP–TYR complex. All TPs survive the binding pose during the whole simulation time. Then, the binding free energies of each functional group with tyrosinase were evaluated using MM/PBSA method, followed by decomposing into four energy components, *i.e.*, Δ*E*_vdW_, Δ*E*_ele_, Δ*G*_nonpolar_ and Δ*G*_polar_. The entropic contributions (−*T*Δ*S*) were calculated using normal mode analysis^43^ based on five conformations. Table 3 lists the results of free energy calculations. It can be observed that the ring B for three TPs with the lower binding free energies has higher proneness to closely contact with the catalytic center of tyrosinase than other functional groups. The order of binding free energies of catechin, ECG and EGCG is highly consistent with their associated constants (*K*_A_) determined by fluorescence quenching. Although EGCG possesses the highest binding ability with tyrosinase, the weakest ability of inactivating tyrosinase than both catechin and ECG results in its lowest tyrosinase inhibitory activity. It was obvious that ECG with the pyrogallol group (ring G) exhibited much stronger binding with tyrosinase than catechin due to the lower binding free energies. This is consistent with the measured inhibitory activity. The larger van der Waals contribution for binding free energy of ECG compared with catechin also explained why the pyrogallol group could enhance the tyrosinase inhibitory activity of TPs very well. The weaker binding ability of catechin with tyrosinase than ECG may be the main reason of its lower inactivation efficiency against tyrosinase. In addition, it can be noted that the pyrogallol group (ring G) also enhances the binding free energy or associated constants (*K*_A_) of EGCG with tyrosinase, but EGCG has weaker tyrosinase inhibitory activity than catechin due to the lack of the catechol group on EGCG, which gives rise to the inactivation of tyrosinase. Moreover, we also found that ring B of ECG and EGCG had higher possibility to closely contact with the catalytic center of tyrosinase than ring G, indicating that their tyrosinase inhibitory activities mainly originated from the contributions of ring B. Further, geometrically, the orientation of ring B of ECG and catechin was approximately orthogonal to the line connecting the coupling copper ions with ∼90° included angles (as shown in Fig. 6), indicating both of them could present in the optimal orientation required by Quintox mechanism,^66^ which results in the inactivation of tyrosinase, while the ring B of EGCG with lowest inhibitory activity possesses unfavorable geometric orientation for the cresolase-like pathway. This further confirms that EGCG inhibits tyrosinase activity by mainly competing with or delaying the oxidation of substrate.

**Table 3:** Binding free energy (kcal mol^−1^) and the energy components for catechin, ECG and EGCG with tyrosinase and their orientations^a^

Complexes		Δ*E*_vdW_	Δ*E*_ele_	Δ*G*_polar_	Δ*G*_nonpolar_	−*T*Δ*S*	Δ*G*_bind_
TPs	Binding group						
Catechin	Ring A	−26.63	11.16	−15.43	−3.66	22.21	−12.35
	Ring B	−22.97	6.99	−14.01	−3.66	21.26	−12.39
ECG	Ring A	−40.63	4.76	−9.48	−4.91	26.19	−24.07
	Ring B	−45.58	8.56	−12.05	−5.03	25.25	−28.85
	Ring G	−35.53	5.63	−11.65	−5.25	29.83	−16.97
EGCG	Ring A	−34.07	3.99	−4.48	−4.95	25.15	−14.36
	Ring B	−41.52	3.06	−9.49	−5.02	23.43	−29.54
	Ring G	−36.11	3.60	−8.56	−5.28	34.03	−12.32

a The van der Waals (Δ*E*_vdW_), electrostatic interaction (Δ*E*_ele_), polar solvation free energy (Δ*G*_polar_) and non-polar solvation energy (Δ*G*_nonpolar_) are calculated and decomposed using MM/PBSA method and the change of conformational entropy (−*T*Δ*S*) is evaluated by normal mode analysis. The binding free energy (Δ*G*_bind_) is the total of five energy items.

## 4. Conclusion

In this work, fluorescence spectroscopy, cyclic voltammetry, oximetry and molecular simulations were employed to shed light on the tyrosinase inhibitory mechanisms of three TPs, including catechin, ECG, and EGCG. Their inhibitory activities are in the order of ECG > catechin > EGCG. The catechol group (ring B) is necessary to inhibit tyrosinase activity with high efficient. TPs have potent quenching effect on the intrinsic fluorescence of tyrosinase with static quenching manner and interact with tyrosinase with the molar proportion of 1 : 1. Cyclic voltammetry profiles and oxidization kinetics of tyrosinase suggest that catechin, ECG and EGCG inhibit the activity of tyrosinase, but with different inhibitory mechanisms. The high inhibitory activities of ECG and catechin were accomplished mainly by making tyrosinase inactivate through cresolase-like catalytic pathway, while EGCG is by competing with or delaying the oxidation of substrate mainly through diphenolase-like catalytic pathway. Molecular simulations further verified the speculated two types of inhibitory mechanisms.

## Conflicts of interest

There are no conflicts to declare.

## Supplementary Material

RA-008-C7RA12749A-s001
