# Supplementary material for: Understanding the inhibitory mechanism of tea polyphenols against tyrosinase using fluorescence spectroscopy, cyclic voltammetry, oximetry, and molecular simulations
Source: RSC Adv. 2018 Feb 22;8(15):8310–8. doi: 10.1039/c7ra12749a (PMC9078569; doi:10.1039/c7ra12749a)
Supplement: RA-008-C7RA12749A-s001 [file RA-008-C7RA12749A-s001.pdf]

Electronic Supplementary Material (ESI) for RSC Advances.
This journal is © The Royal Society of Chemistry 2018

Supporting Information

Understanding the Inhibitory Mechanism of Tea Polyphenols against Tyrosinase Using Fluorescence Spectroscopy, Cyclic Voltammetry, Oximetry, and Molecular Simulations

Haifeng Tang[1,3,5], Fengchao Cui[1*], Haijuan Li[2] ,Qingrong Huang[4], Yunqi Li[1*]

1.Key Laboratory of Synthetic Rubber, Changchun Institute of Applied Chemistry (CIAC), Chinese Academy of Sciences, Changchun 130022, P. R. China.

2.State Key Laboratory of Electroanalytical Chemistry, Changchun Institute of Applied Chemistry (CIAC), Chinese Academy of Sciences, Changchun, Jilin 130022, P.R. China

3. School of Life Science, Jilin University, Changchun 130012, P. R. China.

4. Department of Food Science, Rutgers University, 65 Dudley Road, New Brunswick, NJ 08901, United States

5. University of Chinese Academy of Sciences, Beijing 100049, P.R. China.

∗ Fengchao Cui.
E-mail address: fccui@ciac.ac.cn

∗Yunqi Li.
E-mail address: yunqi@ciac.ac.cn

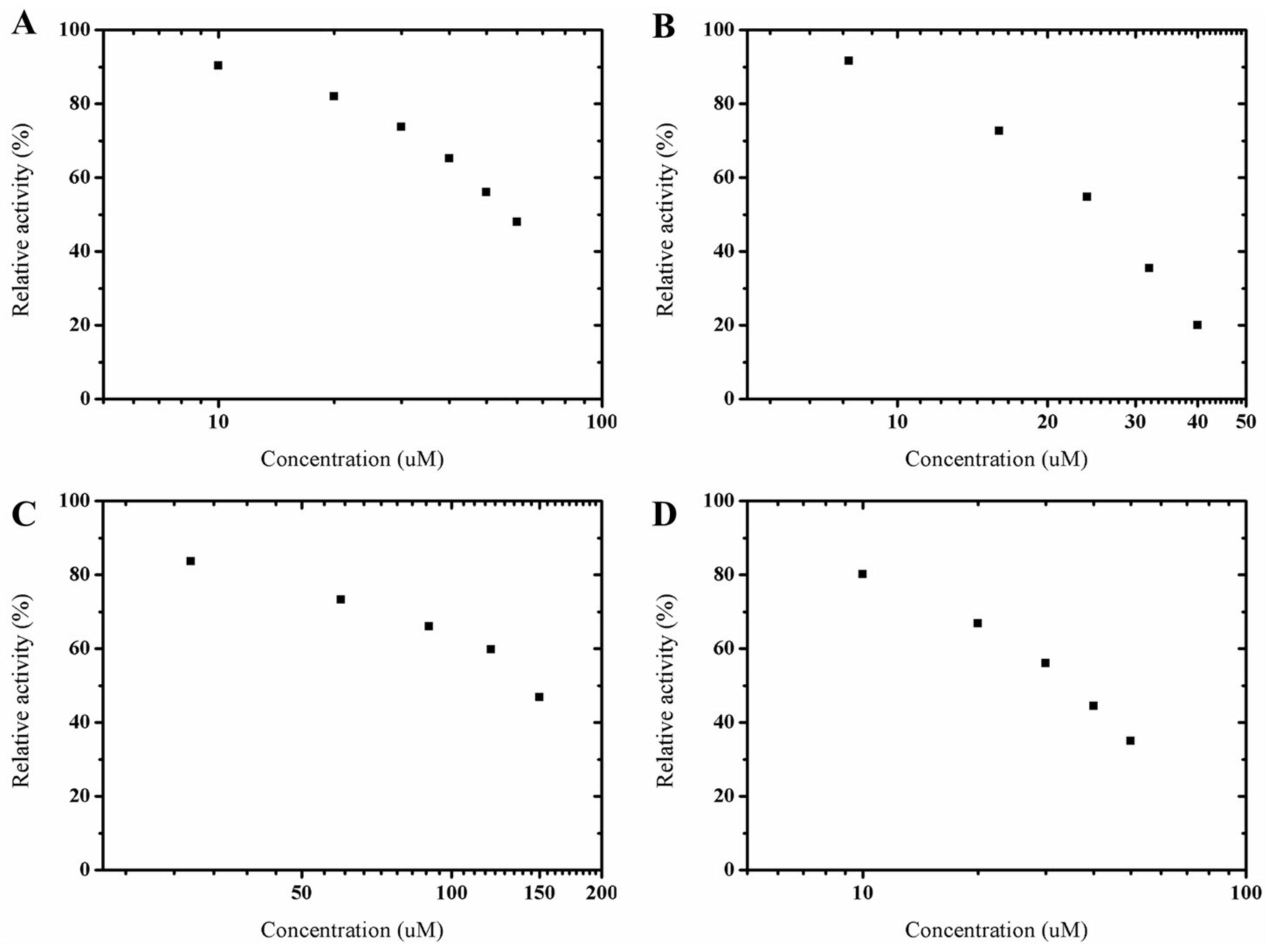

Figure 1. Tea Polyphenols, i.e. catechin (A), ECG (B), EGCG (C) and kojic acid (D) inhibit tyrosinase with dose-dependent manner.

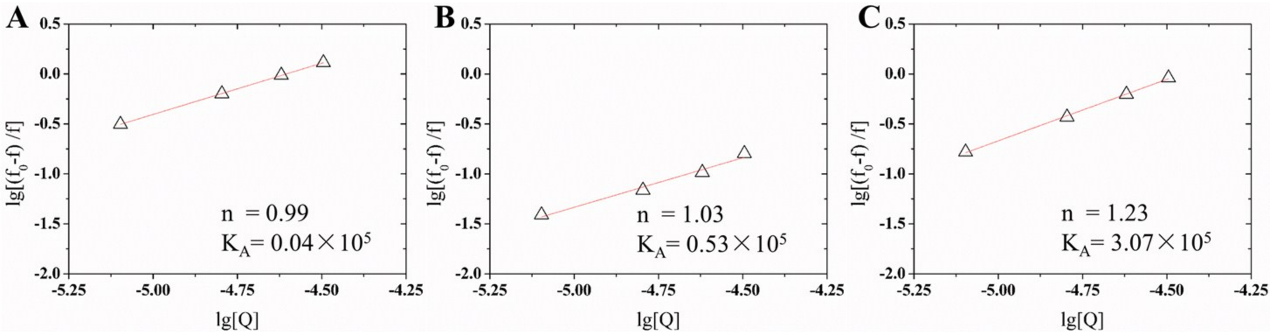

Figure 2. The binding number of TPs with tyrosinase obtained by fitting with equation 2 approximately equals to 1.

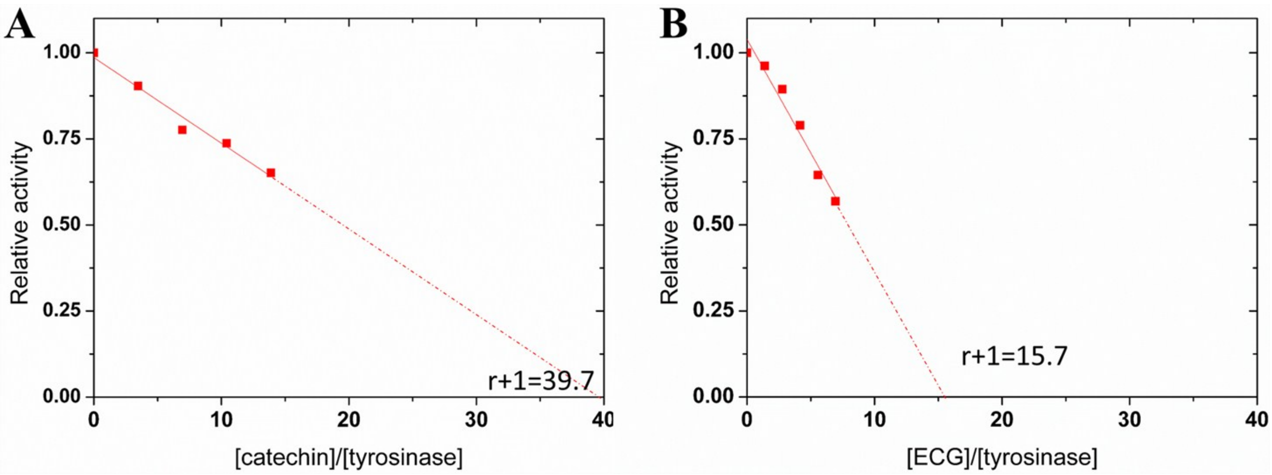

Figure 3. The partition ratio (r) of catechin (A) and ECG (B) is 38.7 and 14.7, respectively. The relative active of tyrosinase is negatively correlate with the molar partition of inhibitor and tyrosinase, and the value of (r+1) can be evaluated from the intercept on abscissa of correlation equation.

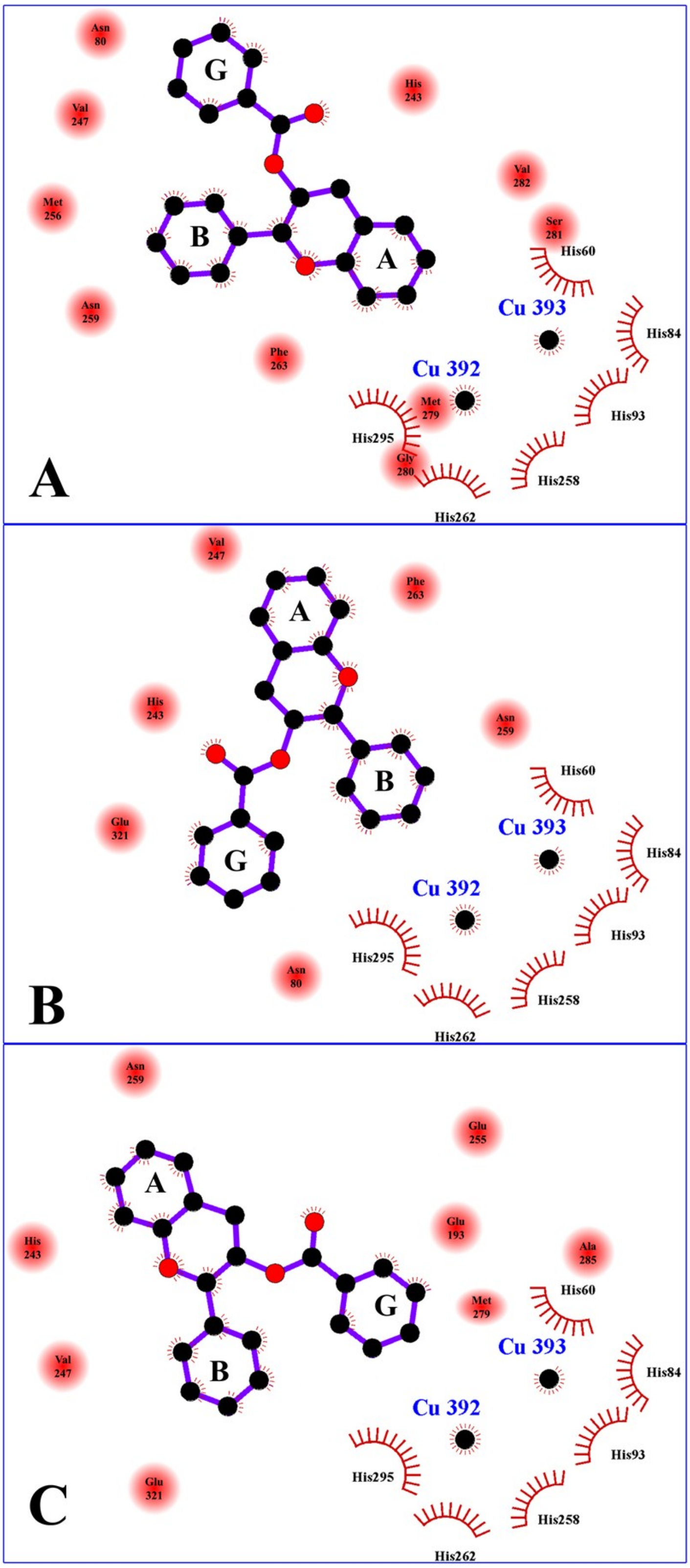

Figure 4. The possible binding poses of TPs coordinating with the bi-copper ions in the active center of tyrosinase. A and B represent ring A and B facing the bi-copper ions for catechin (excluding ring G) and ECG, respectively; C is the ring G orientating to active center for ECG and EGCG, respectively.
